# Perceived Social Support, Study-Related Stress, and Depressive Symptoms in Saudi Medical Students: A Cross-Sectional Study

**DOI:** 10.3390/healthcare14131816

**Published:** 2026-06-23

**Authors:** Hussain Nuri Alali, Rawan Salem Alkhammas, Fatimah Abdullah Alessa, Khalid Jafar Alqadhib, Abdulhakim Ibrahim Alabdullah, Majd Khalid Al Dhailan, Abdullah Almaqhawi

**Affiliations:** 1 Department of Family Medicine and Community, College of Medicine, King Faisal University, Al-Ahsa 36362, Saudi Arabia; 221421582@student.kfu.edu.sa (H.N.A.); 221436592@student.kfu.edu.sa (R.S.A.); 220025195@student.kfu.edu.sa (F.A.A.); 220006998@student.kfu.edu.sa (M.K.A.D.); 2 Academy of Family Medicine, Al-Ahsa Health Cluster, Al-Ahsa 31982, Saudi Arabia; keeld2000@hotmail.com; 3 King Fahad Hospital Hofuf, Al Ahsa Health Cluster, Al-Ahsa 31982, Saudi Arabia; hakeem12121@gmail.com

**Keywords:** medical students, stress, depression, anxiety

## Abstract

**Background:** Medical students are at high risk of psychological distress due to academic and personal pressures. This study assessed stress, depression, and associated factors among medical students, with emphasis on social support. **Methods/Material:** A cross-sectional survey was conducted among 367 medical students at King Faisal University using the Perceived Stress Scale (PSS) and Patient Health Questionnaire (PHQ). Data were analyzed using IBM SPSS version 29.0. **Results:** Participants were nearly equally distributed by gender (51.5% females), with a mean age of 22–23 years. The mean corrected PSS-10 score was 20.19 ± 6.21 and the mean PHQ-9 score was 9.45 ± 5.58; 48.2% screened positive for clinically significant depressive symptoms (PHQ-9 ≥ 10). High stress and depressive symptoms were prevalent; 43.1% frequently felt nervous, 44.7% reported hopelessness, and 43.1% endorsed any thoughts of being better off dead or of self-harm on the PHQ-9 screening item. Peer support was associated with significantly lower stress (PSS: 17.77 vs. 21.25, *p* < 0.001) and depression scores (PHQ: 8.09 vs. 11.0, *p* < 0.001), and remained an independent predictor of lower odds of a positive depression screen in adjusted analysis (adjusted OR 0.90, 95% CI 0.83–0.96). Female and pre-clinical students showed poorer psychosocial outcomes (*p* < 0.05). **Conclusions:** Psychosocial distress is common among medical students, particularly females and pre-clinical students. Higher perceived social support, particularly peer support, was associated with lower stress and depressive symptom scores; given the cross-sectional design these associations cannot establish causation, but they support strengthening peer- and faculty-support systems within medical schools. Findings should be interpreted in light of the cross-sectional, single-centre, self-report design and a response below the pre-specified target, which limit causal inference and generalisability.

## 1. Introduction

Mental health is an essential part of overall well-being. It shows how well a person can handle stress, stay productive, and manage daily life [1]. Depression, which is characterized by a persistent feeling of low mood and a lack of interest in enjoyable activities (anhedonia), is one of the most prevalent and serious mental health issues globally [2]. Medical students are particularly susceptible to mental health disorders, and the prevalence of depressive symptoms among medical students is even higher than that of the general population [3,4,5,6]. This has been attributed to academically demanding curricula, emotional stress, and fear of failure [7]. Lifestyle factors such as smoking, dietary habits, sleep disturbances, and pornography have been linked to poor mental health for medical students [8,9,10,11]. A global analysis including 32 meta-analyses revealed that about 32.5% of medical students are affected by depression, and 8.9% have thoughts of or attempted suicidal behaviour [12]. In India, a systematic review revealed that the prevalence of depression among 5944 medical students could reach as high as 50.0% [13]. Similar mental health issues related to stress or depressive symptoms have been reported among medical students in Saudi Arabia by numerous studies [14,15,16]. A study established at King Faisal University (KFU) showed medical professionals experience significant levels of stress, anxiety, and depressive symptoms. In total, 33.6% of participants in the cross-sectional study exhibited depressive symptoms; however, only 16.2% sought mental health services, even though 42.5% said they felt they needed such support [17]. The results showed that stigma toward mental healthcare persists among medical students, reflecting worldwide attitudes on mental health [17,18]. Many students are concerned that seeking help would negatively impact their academic records and reduce their career opportunities, leading to concerns about discrimination [19]. Increasing social support can play a significant role in lowering stigma and encouraging the use of mental health services [20].

Despite the well-documented prevalence and contributing factors of depression among medical students, less attention has been given to the underlying mechanisms that influence the relationship between stressors and depression, as well as the buffering role of social support [21]. This relationship can be explained from a theoretical perspective by using existing theories of stress, such as the Job Demands–Resources (JD-R) model and transactional theories of stress. The JD-R model suggests that job demands (such as workload and performance pressure) are positively linked to psychological strain, but individual and social resources (such as perceived social support) buffer job demands on psychological health outcomes [22]. Similarly, transactional models of stress emphasize that stress outcomes are dependent on the appraisal of demands and the availability of resources to deal with them [23].

Recent research demonstrates that social support, particularly support that is actually received rather than merely perceived as available, may weaken the link between stress and its negative physical and psychological effects [24]. Schmiedl et al. (2022) revealed that the quality of social support, especially received support, significantly neutralised the associations between job demands, cortisol levels, and felt stress [25].

However, in the context of Saudi medical education, there has been little research exploring the relationship between social resources and the relationship between stressors associated with studying and mental health [26]. Given these results, medical students’ mental health is an investment that is expected to have an impact on the type of doctor they become in the future. Furthermore, understanding the role of social resources and study-related stressors in the influence of depressive symptoms among medical students in Saudi Arabia remains crucial. This is a critical gap that needs to be addressed to develop efficient healthcare systems and create prevention programs that focus on improving protective variables rather than reducing stressors. Our study addresses this gap by examining the associations of social resources with study-related stress and depressive symptoms among medical students at KFU.

## 2. Materials and Methods

The research was conducted in conformance with the guidelines outlined in the Strengthening the Reporting of Observational Studies in Epidemiology (STROBE) Statement: guidelines for reporting observational studies (Appendix A).

This cross-sectional study examined the association of perceived social support with study-related stress and depressive symptoms among medical students at the College of Medicine, King Faisal University, Saudi Arabia, between May and August 2025. The estimated eligible population was 1654 students. This study included 367 medical students who completed the link provided via social media for the online English version of the questionnaire [27]. All male and female medical students in the five academic years (n = 1654) were invited to complete the study questionnaire. Informed consent was obtained, and participants were free to withdraw; their privacy was maintained. The responses were voluntary, and consent was taken from the students before starting the questionnaire. The research study used standardized questionnaires and graphical scales to determine the status level. The inclusion criteria were medical students in the College of Medicine, King Faisal University, Saudi Arabia. The exclusion criteria were any participant who could not or didn’t provide informed consent to participate in the study. The sample size of this study was 511, calculated with a confidence level of 95%, a standard deviation of 0.5, and a margin of error of 5%. However, 367 of 511 medical students completed the questionnaire and were included in the final analysis. This study was approved by the Deanship of Scientific Research of King Faisal University, reference number (KFU-REC-2025-MAY-ETHICS3337).

Recruitment and participant flow. Of the 1654 students invited, 367 provided consent and completed the questionnaire and were included in the final analysis, corresponding to a participation of 22.2% of those invited and 71.8% of the pre-specified target of 511. The participant-flow numbers (students invited, consented, and excluded for incomplete responses) are summarised in the STROBE participant-flow diagram provided as Supplementary Figure S1. The questionnaire was administered in English as a single online instrument distributed through institutional student channels on social media; it was self-completed without direct in-person supervision. Participation was voluntary and responses were collected anonymously, with no identifying information recorded, which is particularly important given the sensitive items on suicidal thoughts, sexual desire, and reproductive health. Because recruitment relied on voluntary online response, self-selection and non-response bias are possible and are addressed in the Limitations.

Risk management for safety-critical responses. PHQ-9 item 9 (thoughts of being better off dead or of self-harm) is a screening item and not a diagnosis of suicidality. Because the survey was anonymous, individual follow-up was not possible; all participants were therefore provided, on the final page of the questionnaire, with the contact details of the university counselling service and national mental health helpline numbers and were advised to seek urgent help if distressed. The absence of an individualised referral pathway under anonymous administration is acknowledged as a limitation. The anonymous data-collection procedure, including the provision of counselling and helpline information in place of individualised follow-up for positive responses to PHQ-9 item 9, formed part of the study protocol reviewed and approved by the King Faisal University research ethics committee (KFU-REC-2025-MAY-ETHICS3337).

Certain questions were abbreviated, and some terms were modified to reflect the values and beliefs of the local community. All instruments were administered in English, the language of medical instruction at the institution; no Arabic translation was used. The adapted wording of all items, as administered, is shown in the questionnaire provided in Appendix B, and the adapted questionnaire was pilot-tested on a small group of students for clarity before distribution. Because adaptation can affect the psychometric properties and cross-study comparability of validated instruments, results are interpreted with this caveat.

Demographic data: We collected the following demographic data: university, year, age, gender, and marital status.

Validated scales and questionnaires were used to collect data. These included the Perceived Stress Scale (PSS-10) (Cohen et al., 1983), the Brief Patient Health Questionnaire (PHQ-9) (Kroenke et al., 2001), the Subjective Social Status Scale (Adler et al., 2000), the Dual Identity Scale (Simon et al., 2008), the Group Identification Scale (Sani et al., 2015), and the Multidimensional Scale of Perceived Social Support (MSPSS) (Zimet et al., 1988) [28,29,30,31,32,33] (Appendix B).

Perceived stressors: Perceived stress was evaluated by using the 10-item version of the Perceived Stress Scale (PSS) developed by Cohen et al. [28]. The PSS is a widely utilized tool used to assess individuals’ perceptions of recent stress [34]. The questionnaire consisted of 10 items that addressed aspects perceived during the last month (e.g., “In the last month, how often have you been upset because of something that happened unexpectedly?”). Students had to select their answers on a five-point Likert response scale: how often the respective stressors occurred (from “never” (=0) to “very often” (=4)).

Depressive symptoms: The Patient Health Questionnaire (PHQ-D) [29] was utilized to measure depressive symptoms. The scale refers to the symptoms felt during the last two weeks and consists of nine items (e.g., “Feeling down, depressed, or hopeless”). Students had to indicate the frequency with which they had experienced the respective symptom on a four-point scale ranging from “not at all” (=0) to “almost every day” (=3).

Social resources: Social support was measured by using a questionnaire of social support at the workplace [35]. In the response option to the items, the words superiors and colleagues were replaced with the words professors and fellow students to evaluate social support from professors and fellow students specifically. For instance, one of the items was “My professors offer help when I am under pressure”. Each item had response options on a four-point scale ranging from “not at all” (=1) to “absolutely” (=4).

As an additional social resource, social identity was measured with the Group Identification Scale [32], which is a four-item scale (e.g., “I feel a connection to other human medicine students/dental medical students”) with answers ranging from “strongly disagree” (=1) to “strongly agree” (=7).

Dual identity was assessed with a scale adapted from Simon et al. and Simon and Ruhs [31,36]. This scale was initially developed for people with a migration background who hold more than one nationality. For this study, we adapted the items for use with Human Medicine Students. The scale consists of four items (e.g., “I consider myself a member of both my study program group and the medical faculty”) with a five-point scale ranging from “not true” (=1) to “absolutely true” (=5).

Subjective social status was measured with an item adapted from Adler et al. [30]. Students were presented with a status ladder, where at the top of the ladder (100) are the people who are best off (most money, most education, most respected jobs), and at the bottom (0) are the worst off (least money, least education, least respected jobs or no job). Students were asked to indicate, using an open-ended numerical input from 0 (“low status”) to 100 (“high status”), where they believed people in their study program stood relative to others in society.

Statistical analyses were performed using IBM SPSS Statistics version 29.0.0.0 (IBM Corp., Armonk, NY, USA) and independently verified in Python version 3.12 (Python Software Foundation, Wilmington, DE, USA, pandas (version 2.2.3), statsmodels (version 0.14.4), and SciPy (version 1.14.1)). Descriptive statistics (mean ± SD, median with IQR, and N (%)) were computed for all variables. The PSS-10 was scored as a single validated scale after reverse-coding the four positively worded items (4, 5, 7, 8); scored correctly, internal consistency was good (Cronbach α = 0.79). The PHQ-9 also showed good reliability (α = 0.84). Normality of the total scores was assessed with the Shapiro–Wilk test; both totals deviated from normality (*p* < 0.001), although skew was modest, so parametric tests were used with key findings confirmed by non-parametric tests (Mann–Whitney U, Kruskal–Wallis). Missing data were minimal as completion of all items was required to submit the questionnaire; analyses used complete cases. Group differences by gender and academic stage were examined using independent *t*-tests or one-way ANOVA, with effect sizes (Cohen’s d, η^2^) reported alongside *p*-values. Associations were explored with Pearson or Spearman correlations. Multivariable linear regression identified predictors of psychological distress (defined as the sum of the corrected PSS-10 and PHQ-9 totals). This composite was used as a single global indicator of psychological distress because the two scales were positively correlated and captured related but complementary dimensions of distress; scale-specific analyses and the screening-based logistic model are reported alongside it, and the implications of combining the two constructs are considered in the Limitations. The linear model included perceived professor and peer support as predictors; collinearity was assessed using the variance inflation factor (VIF). Binary logistic regression estimated the adjusted odds of a positive depression screen (PHQ-9 ≥ 10). Given the number of bivariate comparisons, these subgroup analyses are presented as exploratory. A two-sided *p*-value < 0.05 was considered statistically significant.

## 3. Results

Our study included 367 participating medical students (Table 1). Most were aged 22–23 years (170, 46.3%), followed by 20–21 years (97, 26.4%) and 24–25 years (61, 16.6%), while only 7 (1.9%) were 26 years or above. The sample was almost evenly distributed by gender, with females comprising 189 (51.5%) and males 178 (48.5%). Regarding academic year, the largest group was from the 4th year (134, 36.5%), followed by the 5th year (87, 23.7%), with fewer from the 1st (54, 14.7%), 2nd (45, 12.3%), and 3rd year (47, 12.8%). Most students were single (329, 89.6%), while 38 (10.4%) were married. Seventy-five (20.4%) reported taking medication for anxiety, depression, or stress, whereas 292 (79.6%) were not. Item-level response distributions for the PSS-10 and PHQ-9 are provided in Supplementary Tables S1 and S2.

Depressive symptoms were assessed using the PHQ scale. A considerable proportion reported several days of little interest or pleasure in activities (167, 45.5%) and feeling down or hopeless (164, 44.7%), while 91 (24.8%) and 75 (20.4%) experienced these nearly half the days, respectively. Sleep disturbances were frequent, with 147 (40.1%) facing them for several days and 121 (33.0%) nearly half the days or more. Fatigue was notable, as 169 (46.0%) felt tired on several days and 147 (40.0%) reported this on nearly half the days or almost every day. Poor appetite or overeating affected 132 (36.0%) for several days, while 114 (30.9%) reported self-critical thoughts frequently. Concentration difficulties were common, noted by 220 (59.9%) for at least several days. On PHQ-9 item 9, a screening item rather than a clinical diagnosis, 158 (43.1%) endorsed any thoughts of being better off dead or of hurting themselves at some frequency over the preceding two weeks (the full item-level distribution is provided in Supplementary Table S2).

**Figure 1:**
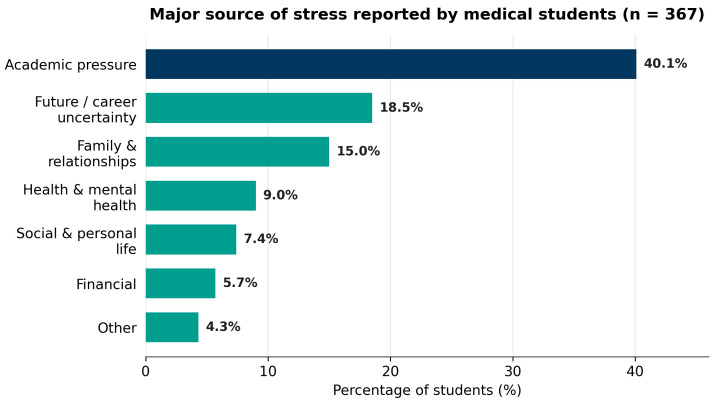
Major Stress Present at this Time of Life in Students (n = 367).

Figure 1 shows the distribution of major stressors reported by medical students. The most prominent source of stress was academic pressure (40.1%), which included exams, grades, research, and medical school demands. This was followed by future and career uncertainty (18.5%) and concerns related to family and relationships (15%). Other reported stressors included health and mental health challenges (9%), issues in social and personal life (7.4%), and financial stress (5.7%). A smaller proportion (4.3%) cited miscellaneous “other” stressors.

Table 2 shows the assessment of anxiety and panic attacks among the participants. In the past four weeks, 121 (33.0%) reported experiencing an anxiety attack, while 246 (67.0%) did not. A history of such episodes was noted by 123 (33.5%), and 90 (24.5%) described attacks occurring unexpectedly. Worry or distress about future attacks was reported by 99 (27.0%). During the last severe episode, 121 (33.0%) experienced somatic symptoms such as palpitations, dizziness, or nausea. Regarding functional impact, 98 (26.7%) found these problems somewhat difficult, 35 (9.5%) very difficult, and 12 (3.3%) extremely difficult. Among female participants (n = 189), 110 (58.2%) reported premenstrual mood disturbances, and 97 (51.3% of female participants) reported that these resolved after menstruation. Responses to the remaining reproductive-health items (childbirth, miscarriage, and difficulty conceiving) appeared implausibly high for a predominantly single student sample, most likely reflecting a skip-logic or item-coding issue that could not be verified from the anonymised dataset. These items are therefore reported descriptively in Supplementary Table S3 only and were not used in any analysis or interpretation.

**Figure 2:**
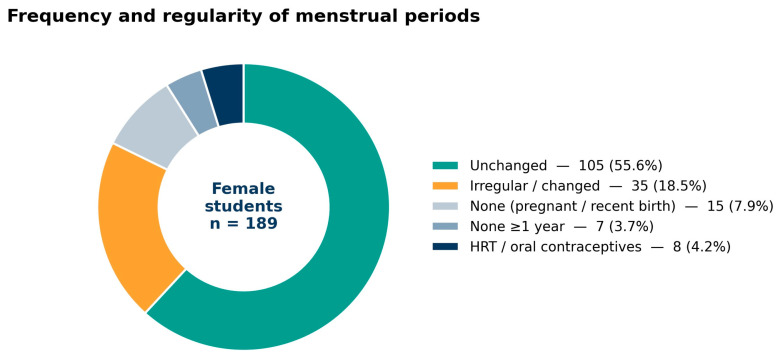
Frequency and regularity of the Periods.

Figure 2 shows the distribution of menstrual health among female students (n = 189). The largest group, 105 (55.6%), reported that their periods remained unchanged. A further 35 (18.5%) experienced irregularity or a change in frequency, duration, or amount. Fifteen (7.9%) had no periods due to pregnancy or recent childbirth, while 7 (3.7%) reported no periods for at least a year, suggesting possible underlying reproductive or hormonal concerns. Eight (4.2%) were taking hormone replacement therapy or oral contraceptives. The remaining 19 students (10.1%) did not report this information. Percentages sum to 100%.

**Figure 3:**
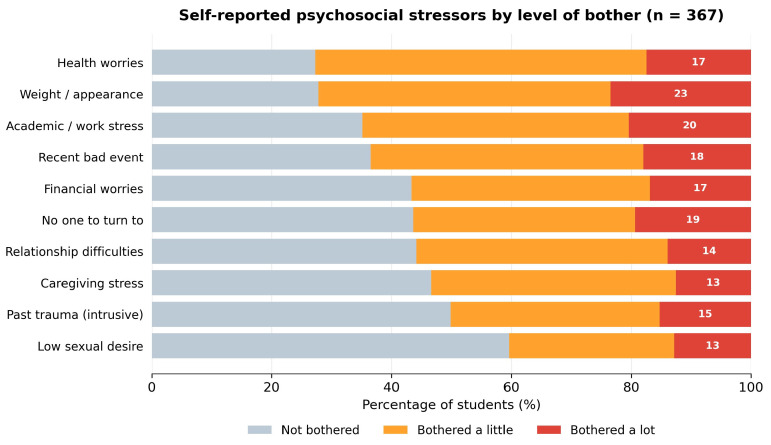
Self-reported psychosocial stressors among study participants, categorized by level of bother.

Figure 3 outlines self-reported psychosocial stressors categorized by level of bother. Worry about health was common, with 203 (55.3%) bothered a little and 64 (17.4%) bothered a lot. Concerns about weight or appearance affected 179 (48.8%) a little and 86 (23.4%) a lot. Reduced sexual desire was noted by 69 (18.8%) a little and 32 (8.7%) a lot. Relationship difficulties were experienced by 154 (42.0%) a little and 51 (13.9%) a lot, while caregiving stress affected 150 (40.9%) a little and 46 (12.5%) a lot. Academic or work-related stress was frequent, with 163 (44.4%) a little and 75 (20.4%) a lot. Financial worries troubled 146 (39.8%) a little and 62 (16.9%) a lot. Notably, 71 (19.3%) felt greatly bothered by a lack of social support. Past adverse events still disturbed 56 (15.3%).

Table 3 shows the perceived social support among medical students from professors and fellow students. Regarding professors, reliance during difficult times was limited, as 144 (39.2%) reported not at all and only 23 (6.3%) absolutely. Professors’ willingness to listen was mostly perceived positively, with 127 (34.6%) responding “mostly,” though 83 (22.6%) felt no support. Help under pressure was reported “a little” by 140 (38.1%) and “mostly” by 99 (27.0%). Feeling supported during challenges was absent for 115 (31.3%), while 48 (13.1%) absolutely agreed. In contrast, fellow students provided greater support. Reliance in difficult times was “mostly” for 122 (33.2%) and “absolutely” for 52 (14.2%). Listening and help were frequently affirmed, with 120 (32.7%) and 145 (39.5%) reporting “mostly.” Notably, 207 (56.4%) felt supported by peers at least “mostly”.

Table 4 shows students’ perceptions of group identification within their study program and dual identity with the wider medical faculty. Identification with peers in the same program was mixed, with 113 (30.8%) disagreeing and 91 (24.8%) strongly disagreeing, while only 86 (23.4%) agreed and 22 (6.0%) strongly agreed. Similarly, feelings of bond and solidarity were low, with 135 (36.8%) and 156 (42.5%) disagreeing, though about one-quarter agreed. A sense of connection was more evenly distributed, with 93 (25.3%) agreeing and 30 (8.2%) strongly agreeing, but 178 (48.5%) remained neutral or disagreed. Regarding dual identity, most students leaned neutral to slightly true: 101 (27.5%) felt moderately connected to both program and faculty, while stronger affirmation (“mostly true” or “absolutely true”) was relatively low, ranging between 19 (5.2%) and 31 (8.4%).

Table 5 shows the association of perceived social resources with study-related stress (PSS scores) among students. Support from professors showed a significant association: those who felt strongly supported reported lower stress, with mean PSS scores decreasing from 21.12 (6.36) among students who felt “not at all” supported to 18.75 (4.37) among those who felt “mostly” supported (*p* = 0.009). Similarly, reliance on professors when facing difficulties revealed lower stress scores for “mostly” (18.74, 3.21) compared to “not at all” (20.64, 5.87), reaching statistical significance (*p* = 0.037). Peer support demonstrated even stronger associations. Students reporting that fellows were willing to listen showed significantly reduced stress, dropping from 21.25 (4.84) in “a little” to 17.77 (5.07) in “absolutely” supported (*p* < 0.001).

Table 6 shows the associations between social resources and depressive symptoms among students based on PHQ scores. Support from professors showed mixed associations. Students who reported professors offering help under pressure had significantly lower depressive symptoms when they felt “absolutely” supported (mean 6.97, SD 5.22) compared to those with “not at all” support (10.38, SD 6.27), reaching significance (*p* = 0.019). However, other professor-related domains, such as reliance and willingness to listen, did not show statistically significant differences. By contrast, support from fellow students was consistently associated with lower depressive symptom scores. Students who could rely on peers had markedly lower PHQ scores, decreasing from 10.69 (SD 5.68) in “not at all” to 7.54 (SD 4.94) in “absolutely” supported (*p* = 0.004). Similarly, peers’ willingness to listen showed a strong association, with scores dropping from 11.00 (SD 5.41) to 8.09 (SD 5.34) (*p* < 0.001).

Table 7 compares psychological distress scores (corrected PSS-10 plus PHQ-9) by gender across levels of perceived social support. Female students consistently reported higher distress scores than males at comparable levels of support. For professor support, significant gender differences were observed: females who could rely on professors reported higher distress scores (28.59, SD 7.52) than males (25.77, SD 6.21) (*p* = 0.014), with similar patterns when professors offered help under pressure (30.73, SD 8.12 vs. 27.49, SD 7.66; *p* = 0.042) and during challenging times (29.06, SD 8.02 vs. 26.47, SD 7.22; *p* = 0.035). Gender differences were even more marked for peer support, with significant differences across all items (*p* < 0.001). For example, distress decreased from 32.33 (SD 9.32) among females reporting no peer reliance to 25.96 (SD 8.71) among those feeling absolutely supported, while males showed lower distress scores throughout (24.79, SD 8.82 with absolute support).

Table 8 compares psychological distress scores by academic stage (pre-clinical versus clinical) across levels of perceived social support. Pre-clinical students consistently reported higher distress scores than clinical students. For example, among students who relied on professors when facing difficulties, distress was higher in pre-clinical students (31.89, SD 5.96) than in clinical students (25.55, SD 6.51) (*p* = 0.024), and among those who felt supported by professors during challenges, pre-clinical students again reported higher distress (33.35, SD 7.57) than clinical peers (26.13, SD 6.99) (*p* = 0.025), with a comparable pattern for professors offering help under pressure (*p* = 0.032). The contrast was sharper for peer support, with significant differences across all domains (*p* < 0.001 to *p* = 0.002). For instance, pre-clinical students who could rely on peers “mostly” reported a mean distress score of 35.29 (SD 7.91), compared with 26.72 (SD 8.56) among clinical students, and among students feeling absolutely supported by peers during challenging times, distress remained higher in pre-clinical (32.80, SD 8.11) than in clinical students (24.26, SD 9.05; *p* < 0.001).

### Total Scale Scores, Severity Categories, and Adjusted Analyses

Total scale scores, internal consistency, severity categories, and the adjusted regression models are presented in Table 9, Table 10, Table 11, Table 12, Table 13, Table 14 and Table 15. The PSS-10 was scored as a single validated scale (reverse-coding items 4, 5, 7, and 8), and all dependent analyses use this score.

After adjustment, female gender and pre-clinical stage independently predicted higher psychological distress. Peer support was associated with lower odds of a positive depression screen, with each additional point lowering the odds by about 10% (adjusted OR 0.90, 95% CI 0.83–0.96, *p* = 0.002), whereas professor support was not independently associated. Given the cross-sectional design, these are associations and do not establish causation.

## 4. Discussion

Good mental health reflects the ability to cope with stress and remain productive, while poor mental health, such as depression, impairs daily functioning [37]. Medical students face elevated risks of stress and depression owing to demanding curricula, social isolation, and lifestyle factors. Previous literature has highlighted persistent mental health challenges in this population, with stigma often preventing students from seeking mental health care. Social support has been associated with lower stress and reduced stigma [38]. This study aimed to explore the association of social resources with stress and depressive symptoms among medical students.

Our findings showed that academic stress was the predominant source of pressure among students (40.1%), followed by career-related uncertainty (18.5%) and family or relationship issues (15%). These results are consistent with the international literature, in which medical students frequently report academic burden as the primary stressor. A study by Di Vincenzo et al. (2024) reported that workload, examinations, and performance pressure are central correlates of psychological distress among medical students [39].

Another study by Rahman et al. (2013) in Saudi Arabia identified the examinations and academic expectations as key stressors for medical students [40]. These findings underscore the universal nature of academic-related stress in medical education across different cultural contexts.

Furthermore, nearly half of the participants frequently felt nervous and stressed, and one in four students often perceived difficulties piling up beyond their control. These findings mirror reports from Nepal, where more than one-fourth of medical students consistently reported moderate-to-high stress levels (Bhandari et al. 2025) [41]. Such consistent results suggested that the medical education’s competitive environment fosters stress globally, despite variations in curricula or support systems.

Depressive symptoms were highly prevalent, with nearly half of the students reporting little interest in activities, sleep disturbances, and fatigue. In addition, 43.1% of participants endorsed PHQ-9 item 9 (thoughts of being better off dead or of self-harm) at some frequency; this represents a screening-level indication of thoughts of death or self-harm rather than clinically established suicidal ideation, and the proportion should be interpreted with this distinction in mind. Previous literature consistently documents elevated depression rates among medical students. A study by AlJaber et al. (2020) reported a pooled prevalence of depression or depressive symptoms of 51.5%, higher than that observed in our study [42]. Another Saudi study by Alyoubi et al. (2025) reported depressive symptoms in 25.6% of medical students [43]. Together with our findings, these reports suggest that Saudi medical students carry a burden of depressive symptoms comparable to that of their international peers.

Students reported stronger support from peers than from professors, and in bivariate analyses peer support was consistently associated with lower stress and depressive symptom scores. This pattern was only partially preserved after adjustment. In the multivariable models, peer support remained independently associated with lower odds of a positive depression screen (adjusted OR 0.90, 95% CI 0.83 to 0.96), whereas its association with the composite distress score was attenuated and no longer statistically significant once gender, academic stage, age, and marital status were taken into account. The bivariate associations may therefore partly reflect confounding by these characteristics, and the findings should be regarded as hypothesis-generating associations rather than evidence of a protective effect. This echoes the findings of the study by König et al. (2023), which underscored the buffering role of peer networks against stress [44]. Professor support showed weaker and less consistent bivariate associations and was not independently associated with either outcome after adjustment. This contrasts with findings from Western institutions, where structured mentorship has shown stronger associations with wellbeing among medical and paramedical students (Jordan et al. 2019, Ohue et al. 2024) [45,46]. The weaker associations observed for professor support in our context may reflect hierarchical barriers or limited faculty-student engagement in Saudi medical schools.

Gender differences were prominent, with female students consistently reporting higher stress and depressive symptom scores than males, and female gender remained independently associated with higher psychological distress in the adjusted models. This finding aligns with the study by Mirza et al. (2021), which found that female medical students have a higher risk of depression and anxiety [47]. The cultural expectations and the gender-specific stressors may exacerbate this disparity in the Saudi context. The national literature also echoes this trend with Almalki et al. (2019) noting significantly higher depression rates among female students in Riyadh medical colleges [48].

Academic stage was another correlate of distress. Pre-clinical students reported higher distress than their clinical peers, and clinical stage remained independently associated with lower distress and with lower odds of a positive depression screen in the adjusted models. These findings are consistent with the study by Akhtar et al. (2023), in which pre-clinical students faced greater adjustment stress when transitioning from secondary education into demanding medical curricula [49]. Conversely, the clinical students often benefit from patient interaction and professional identity formation, which may foster resilience. Locally, a Saudi study by Khlaiwi et al. (2025) similarly found higher stress among pre-clinical students, validating our results [50].

### 4.1. Limitation

There are several limitations of this study. The cross-sectional design of this study prevented the establishment of causality between social resources and mental health outcomes. The self-reported measures may be subject to the recall bias and social desirability bias, particularly concerning sensitive topics like depressive symptoms or reproductive health. The single-center setting at King Faisal University limited the generalizability to other institutions or regions. Additionally, the unmeasured confounding factors, such as lifestyle habits, socioeconomic background, or family dynamics, may have influenced the outcomes. Finally, the reliance on the questionnaires restricted the deeper qualitative insights into students’ lived experiences. A further limitation is the response rate: only 367 of 1654 invited students participated (22.2% of those invited and 71.8% of the pre-specified target of 511). Because recruitment relied on voluntary online responses, the sample may not be representative of the wider student body, and self-selection and non-response bias cannot be excluded. Students experiencing greater distress may have been either more inclined to take part, because the topic was personally relevant, or less inclined, owing to low energy, avoidance, or concerns about disclosure; either pattern would bias the prevalence estimates in an unknown direction. The prevalence figures should therefore be interpreted with caution. In addition, the primary distress outcome combined the corrected PSS-10 and PHQ-9 into a single composite. Although the two scales were positively correlated, they measure related but distinct constructs, and combining them may obscure scale-specific associations; for this reason the scale-specific and screening-based analyses are reported alongside the composite. Finally, several validated instruments were linguistically and culturally adapted and administered in English only, which may have affected their psychometric properties and cross-study comparability.

### 4.2. Future Directions and Implications

There is an urgent need to prioritize mental health support for medical students by strengthening the social resources, especially the peer and faculty support systems. Universities should integrate structured mentorship programs, counseling services, and peer-support initiatives to reduce stress and depressive symptoms while promoting resilience. Faculty training to foster approachable and supportive academic environments is also crucial. The future research should employ the longitudinal and multi-center designs to confirm causal relationships and enhance generalizability. Moreover, the qualitative studies could provide richer insights into students’ lived experiences, which will guide the tailored interventions for sustainable mental health improvement.

## 5. Conclusions

Our study showed a high burden of stress, depressive symptoms, and anxiety among medical students, with academic pressure emerging as the leading reported stressor; clinically significant depressive symptoms (PHQ-9 ≥ 10) were present in 48.2% of participants. Significant associations were found between social support and psychological distress, with peer support more consistently associated with lower stress and depressive symptom scores than professor support. Female gender and pre-clinical stage were independently associated with poorer outcomes. Because the design was cross-sectional, these findings describe associations rather than causal effects, but they support structured mental health programs, early screening, and enhanced peer- and faculty-support systems to address psychological distress and promote student well-being.

## Figures and Tables

**Table 1:** Sociodemographic Features of participants (n = 367).

		Frequency N (%)
Age	18–19	32 (8.7%)
	20–21	97 (26.4%)
	22–23	170 (46.3%)
	24–25	61 (16.6%)
	26 or Above	7 (1.9%)
Gender	Female	189 (51.5%)
	Male	178 (48.5%)
Academic Year	1st	54 (14.7%)
	2nd	45 (12.3%)
	3rd	47 (12.8%)
	4th	134 (36.5%)
	5th	87 (23.7%)
Marital Status	Single	329 (89.6%)
	Married	38 (10.4%)
Are you taking any medication for anxiety, depression, or stress?	No	292 (79.6%)
	Yes	75 (20.4%)

**Table 2:** Assessment of Anxiety and Panic Attacks (n = 367).

		Frequency Number (%)
In the last 4 weeks, have you had an anxiety attack (suddenly feeling fear or panic)?	No	246 (67.0%)
	Yes	121 (33.0%)
Has this ever happened before?	No	90 (24.5%)
	Yes	123 (33.5%)
Do some of these attacks come suddenly out of the blue—that is, in situations where you don’t expect to be nervous or uncomfortable?	No	119 (32.4%)
	Yes	90 (24.5%)
Do these attacks bother you a lot, or are you worried about having another attack?	No	107 (29.2%)
	Yes	99 (27.0%)
During your last bad anxiety attack, did you have symptoms like shortness of breath, sweating, your heart racing or pounding, dizziness or faintness, tingling or numbness, or nausea or upset stomach?	No	81 (22.1%)
	Yes	121 (33.0%)
If you checked off any problems on this questionnaire so far, how difficult have these problems made it for you to do your work, take care of things at home, or get along with other people?	Extremely difficult	12 (3.3%)
	Not difficult at all	60 (16.3%)
	Somewhat difficult	98 (26.7%)
	Very difficult	35 (9.5%)

**Table 3:** Perceived social support by students from professors and fellow students.

	Not at All	A Little	Mostly	Absolutely
Social Support by Professors				
I can rely on my professors when things get difficult during my studies.	144 (39.2%)	118 (32.2%)	82 (22.3%)	23 (6.3%)
My professors are willing to listen to my academic problems.	83 (22.6%)	123 (33.5%)	127 (34.6%)	34 (9.3%)
My professors offer help when I am under pressure.	93 (25.3%)	140 (38.1%)	99 (27.0%)	35 (9.5%)
I feel supported by my professors during challenging times.	115 (31.3%)	117 (31.9%)	87 (23.7%)	48 (13.1%)
Social Support by Fellows				
I can rely on my fellow students when things get difficult during my studies.	106 (28.9%)	87 (23.7%)	122 (33.2%)	52 (14.2%)
My fellow students are willing to listen to my academic problems.	80 (21.8%)	103 (28.1%)	120 (32.7%)	64 (17.4%)
My fellow students offer help when I am under pressure.	70 (19.1%)	84 (22.9%)	145 (39.5%)	68 (18.5%)
I feel supported by my fellow students during challenging times.	82 (22.3%)	78 (21.3%)	130 (35.4%)	77 (21.0%)

**Table 4:** Distribution of responses on group identification and dual identity, evaluating students’ perceived affiliation with their study program and the medical faculty.

	Strongly Disagree	Disagree	Neutral	Agree	Strongly Agree
I identify strongly with other students in my study program.	91 (24.8%)	113 (30.8%)	55 (15.0%)	86 (23.4%)	22 (6.0%)
I feel a bond with other students in my study program.	57 (15.5%)	135 (36.8%)	45 (12.3%)	103 (28.1%)	27 (7.4%)
I feel solidarity with other students in my study program.	50 (13.6%)	156 (42.5%)	58 (15.8%)	79 (21.5%)	24 (6.5%)
I feel a connection to other students in my study program.	60 (16.3%)	118 (32.2%)	66 (18.0%)	93 (25.3%)	30 (8.2%)
	Not True	Slightly True	Moderately True	Mostly True	Absolutely True
I feel connected to the students in my study program as well as to the medical faculty.	97 (26.4%)	98 (26.7%)	101 (27.5%)	52 (14.2%)	19 (5.2%)
I identify both with my specific program (e.g., human medicine) and with the wider medical faculty.	66 (18.0%)	112 (30.5%)	112 (30.5%)	58 (15.8%)	19 (5.2%)
I consider myself a member of both my study program and the medical faculty.	59 (16.1%)	89 (24.3%)	134 (36.5%)	54 (14.7%)	29 (7.9%)
I feel that I belong equally to my program group and to the overall medical faculty at my university.	66 (18.0%)	100 (27.2%)	96 (26.2%)	72 (19.6%)	31 (8.4%)

**Table 5:** Association of perceived social support with perceived stress (corrected PSS-10) among students.

	Not at All	A Little	Mostly	Absolutely	*p*-Value
Support From Professor					
I can rely on my professors when things get difficult during my studies.	20.64 (5.87)	20.16 (4.62)	18.74 (3.21)	18.91 (6.72)	0.037
My professors are willing to listen to my academic problems.	20.89 (5.86)	20.01 (4.58)	19.31 (4.86)	19.88 (5.48)	0.180
My professors offer help when I am under pressure.	20.88 (6.14)	20.01 (4.63)	19.32 (4.52)	19.03 (5.03)	0.122
I feel supported by my professors during challenging times.	21.12 (6.36)	19.92 (4.06)	18.75 (4.37)	19.42 (4.60)	0.009
Support from Fellow Students					
I can rely on my fellow students when things get difficult during my studies.	20.22 (5.52)	21.16 (4.25)	19.80 (4.86)	17.77 (5.37)	0.002
My fellow students are willing to listen to my academic problems.	20.71 (5.01)	21.25 (4.84)	19.50 (4.95)	17.77 (5.07)	<0.001
My fellow students offer help when I am under pressure.	20.23 (5.74)	21.13 (4.17)	19.79 (4.89)	18.56 (5.52)	0.018
I feel supported by my fellow students during challenging times.	20.71 (5.34)	21.32 (4.72)	19.65 (4.57)	18.27 (5.54)	<0.001

**Table 6:** Association of perceived social support with depressive symptoms (PHQ-9) among students.

	Not at All	A Little	Mostly	Absolutely	*p*-Value
Support From Professor					
I can rely on my professors when things get difficult during my studies.	10.11 (5.61)	9.51 (5.63)	8.20 (5.22)	9.48 (6.00)	0.103
My professors are willing to listen to my academic problems.	10.27 (6.07)	9.81 (5.42)	8.80 (5.54)	8.56 (4.87)	0.182
My professors offer help when I am under pressure.	10.38 (6.27)	9.25 (5.26)	9.74 (5.27)	6.97 (5.22)	0.019
I feel supported by my professors during challenging times.	10.26 (6.25)	8.97 (5.11)	8.79 (4.91)	9.85 (6.03)	0.193
Support from Fellow Students					
I can rely on my fellow students when things get difficult during my studies.	10.69 (5.68)	9.86 (5.60)	8.89 (5.51)	7.54 (4.94)	0.004
My fellow students are willing to listen to my academic problems.	10.49 (6.33)	11.00 (5.41)	8.15 (4.86)	8.09 (5.34)	<0.001
My fellow students offer help when I am under pressure.	10.61 (5.98)	10.61 (5.73)	8.91 (5.07)	7.97 (5.62)	0.005
I feel supported by my fellow students during challenging times.	11.04 (6.10)	10.26 (5.71)	8.70 (4.81)	8.21 (5.68)	0.002

**Table 7:** Psychological distress scores by gender across levels of perceived social support.

Variable	Gender	Not at All	A Little	Mostly	Absolutely	*p*-Value
Support From Professor						
I can rely on my professors when things get difficult	Female	32.20 (10.03)	32.87 (8.36)	28.59 (7.52)	29.23 (11.77)	0.014
	Male	28.40 (9.36)	27.06 (8.75)	25.77 (6.21)	27.30 (10.24)	
My professors are willing to listen to my academic problems	Female	33.78 (11.01)	32.30 (8.79)	29.05 (8.47)	30.94 (7.40)	0.113
	Male	26.97 (8.26)	27.39 (7.88)	27.24 (9.10)	26.22 (8.61)	
My professors offer help when I am under pressure	Female	33.05 (11.22)	32.09 (8.49)	30.73 (8.12)	26.00 (6.82)	0.042
	Male	28.00 (9.66)	26.81 (8.29)	27.49 (7.66)	26.00 (9.13)	
I feel supported by my professors during challenging times	Female	33.03 (11.81)	31.53 (6.76)	29.06 (8.02)	31.07 (8.60)	0.035
	Male	29.00 (9.64)	26.31 (8.51)	26.47 (7.22)	26.95 (7.94)	
Support From Fellows						
I can rely on my fellow students when things get difficult	Female	32.33 (9.32)	33.09 (8.67)	31.43 (9.76)	25.96 (8.71)	<0.001
	Male	28.74 (8.79)	27.79 (7.83)	26.85 (8.25)	24.79 (8.82)	
My fellow students are willing to listen to my academic problems	Female	32.84 (9.84)	32.76 (9.77)	30.98 (8.40)	27.24 (8.57)	<0.001
	Male	28.47 (9.49)	31.57 (7.15)	24.83 (7.53)	24.97 (8.39)	
My fellow students offer help when I am under pressure	Female	33.10 (10.30)	31.95 (8.59)	31.18 (8.80)	29.19 (10.70)	<0.001
	Male	27.66 (9.11)	31.34 (7.67)	26.63 (8.03)	24.78 (8.36)	
I feel supported by my fellow students during challenging times	Female	33.40 (9.74)	31.63 (9.70)	30.96 (8.02)	29.73 (10.15)	<0.001
	Male	29.16 (9.02)	31.50 (7.96)	26.50 (7.73)	23.48 (7.94)	

**Table 8:** Psychological distress scores by academic stage across levels of perceived social support.

Variable	Academic Stage	Not at All	A Little	Mostly	Absolutely	*p*-Value
Support From Professor						
I can rely on my professors when things get difficult	Pre-Clinical	30.95 (8.11)	33.41 (9.02)	31.89 (5.96)	29.86 (7.20)	0.024
	Clinical	30.67 (10.57)	28.15 (8.62)	25.55 (6.51)	27.75 (12.37)	
My professors are willing to listen to my academic problems	Pre-Clinical	32.14 (10.37)	31.50 (6.73)	32.27 (7.84)	31.46 (8.03)	0.103
	Clinical	30.82 (10.64)	29.23 (9.22)	26.65 (8.71)	26.57 (8.07)	
My professors offer help when I am under pressure	Pre-Clinical	33.50 (10.27)	30.58 (6.59)	35.48 (7.12)	27.69 (7.24)	0.032
	Clinical	30.56 (11.08)	28.68 (9.54)	27.33 (7.36)	25.00 (8.48)	
I feel supported by my professors during challenging times	Pre-Clinical	31.72 (8.35)	31.00 (7.58)	33.35 (7.57)	32.59 (9.21)	0.025
	Clinical	31.27 (11.94)	27.96 (8.18)	26.13 (6.99)	27.45 (7.62)	
Support From Fellows						
I can rely on my fellow students when things get difficult	Pre-Clinical	31.32 (6.28)	30.80 (10.07)	35.29 (7.91)	27.83 (5.44)	<0.001
	Clinical	30.71 (10.38)	31.11 (8.18)	26.72 (8.56)	24.55 (9.39)	
My fellow students are willing to listen to my academic problems	Pre-Clinical	30.86 (9.34)	34.05 (8.34)	31.08 (7.30)	29.40 (5.47)	<0.001
	Clinical	31.32 (10.14)	31.24 (8.84)	26.70 (8.58)	24.78 (8.96)	
My fellow students offer help when I am under pressure	Pre-Clinical	31.27 (8.72)	32.12 (8.74)	31.37 (6.87)	33.41 (8.73)	0.002
	Clinical	30.65 (10.78)	31.58 (8.10)	27.85 (9.02)	24.24 (9.05)	
I feel supported by my fellow students during challenging times	Pre-Clinical	31.76 (8.62)	32.08 (8.72)	31.21 (7.12)	32.80 (8.11)	<0.001
	Clinical	31.74 (10.13)	31.34 (9.23)	27.53 (8.24)	24.26 (9.05)	

**Table 9:** Total scale scores, internal consistency, and normality (n = 367).

Scale	n	Mean ± SD	Median (IQR)	Range	Cronbach α	Shapiro *p*
PSS-10 (corrected)	367	20.19 ± 6.21	20 (17–24)	5–39	0.79	<0.001
PHQ-9	367	9.45 ± 5.58	9 (5–14)	0–25	0.84	<0.001

**Table 10:** PHQ-9 depression severity using validated cut-offs (n = 367).

PHQ-9 Severity	n	%
None/minimal (0–4)	84	22.9%
Mild (5–9)	106	28.9%
Moderate (10–14)	107	29.2%
Moderately severe (15–19)	54	14.7%
Severe (20–27)	16	4.4%
Clinically significant (PHQ-9 ≥ 10)	177	48.2%

**Table 11:** Perceived stress categories using the corrected PSS-10 (n = 367).

PSS-10 Category	n	%
Low (0–13)	44	12.0%
Moderate (14–26)	271	73.8%
High (27–40)	52	14.2%

**Table 12:** Distribution of PHQ-9 item 9 (screening item, not a clinical diagnosis; n = 367).

Response to Thoughts of Being Better Off Dead/Self-Harm	n	%
Not at all	209	56.9%
Several days	89	24.3%
Nearly half the days	45	12.3%
Nearly every day	24	6.5%
Any endorsement (>not at all)	158	43.1%

**Table 13:** Multivariable linear regression on psychological distress (corrected PSS-10 + PHQ-9; R^2^ = 0.176, adjusted R^2^ = 0.162, model *p* < 0.001; all VIF < 1.8).

Predictor	B	β	95% CI (B)	*p*	VIF
Age band	−0.51	−0.05	−1.85 to +0.84	0.460	1.71
Clinical stage (vs. pre-clinical)	−2.80	−0.13	−5.48 to −0.11	0.041	1.67
Male (vs. female)	−6.86	−0.35	−8.82 to −4.90	<0.001	1.13
Married (vs. single)	−0.15	−0.00	−3.32 to +3.02	0.925	1.10
Professor support (0–12)	−0.32	−0.10	−0.65 to +0.02	0.063	1.22
Peer support (0–12)	−0.13	−0.05	−0.43 to +0.16	0.373	1.26

**Table 14:** Binary logistic regression—adjusted odds of a positive depression screen (PHQ-9 ≥ 10); model pseudo-R^2^ = 0.084.

Predictor	Adjusted OR	95% CI	*p*
Age band	0.78	0.57 to 1.07	0.122
Clinical stage (vs. pre-clinical)	0.53	0.28 to 0.99	0.047
Male (vs. female)	0.54	0.34 to 0.85	0.008
Married (vs. single)	1.01	0.48 to 2.11	0.979
Professor support (0–12)	1.00	0.92 to 1.08	0.985
Peer support (0–12)	0.90	0.83 to 0.96	0.002

**Table 15:** Effect sizes for headline associations.

Comparison	Groups	Test	*p*	Effect Size
Gender → PSS-10	F vs. M	*t*-test	<0.001	d = 0.87 (large)
Gender → PHQ-9	F vs. M	*t*-test	<0.001	d = 0.41 (small–moderate)
Gender → distress	F vs. M	*t*-test	<0.001	d = 0.78 (medium–large)
Peer willing to listen → PHQ-9	4 cats	ANOVA	<0.001	η^2^ = 0.057 (medium)
Peer willing to listen → PSS-10	4 cats	ANOVA	0.004	η^2^ = 0.036 (small–medium)

## Data Availability

The data presented in this study are not publicly available due to ethical and privacy considerations related to the sensitive nature of mental health data collected from students. De-identified data may be made available from the corresponding author upon reasonable request and with approval from the relevant institutional ethics committee.

## Supplementary Materials

The following supporting information can be downloaded at https://www.mdpi.com/article/10.3390/healthcare14131816/s1.

## Abbreviations

The following abbreviations are used in this manuscript:

MSPSS	Multidimensional Scale of Perceived Social Support
PHQ	Patient Health Questionnaire
PHQ-9	Patient Health Questionnaire—9
PSS	Perceived stress scale
PSS-10	Perceived stress scale—10
SD	Standard Deviation
STROBE	Strengthening the Reporting of Observational Studies in Epidemiology

## Appendix A

**Table A1:** STROBE Statement—checklist of items that should be included in reports of observational studies.

	Item No.	Recommendation	Page No.	Relevant Text from Manuscript
Title and abstract	1	(a) Indicate the study’s design with a commonly used term in the title or the abstract	1	Title and abstract
		(b) Provide in the abstract an informative and balanced summary of what was done and what was found	1	Abstract
Introduction				
Background/rationale	2	Explain the scientific background and rationale for the investigation being reported	2–3	Background, Paragraph 1–4
Objectives	3	State specific objectives, including any prespecified hypotheses	3	Background, Paragraph 4
Methods				
Study design	4	Present key elements of study design early in the paper	3	Methods, Paragraph 1
Setting	5	Describe the setting, locations, and relevant dates, including periods of recruitment, exposure, follow-up, and data collection	2–5	Methods, Paragraph 1, 2, 5
Participants	6	(a) Cohort study—Give the eligibility criteria, and the sources and methods of selection of participants. Describe methods of follow-up Case–control study—Give the eligibility criteria, and the sources and methods of case ascertainment and control selection. Give the rationale for the choice of cases and controls Cross-sectional study—Give the eligibility criteria, and the sources and methods of selection of participants	3	Methods, Paragraph 3
		(b) Cohort study—For matched studies, give matching criteria and number of exposed and unexposed Case–control study—For matched studies, give matching criteria and the number of controls per case		
Variables	7	Clearly define all outcomes, exposures, predictors, potential confounders, and effect modifiers. Give diagnostic criteria, if applicable	4–5	Methods, Paragraph 3
Data sources/measurement	8 *	For each variable of interest, give sources of data and details of methods of assessment (measurement). Describe comparability of assessment methods if there is more than one group	*4*	*Methods, Paragraph 3*–*5*
Bias	9	Describe any efforts to address potential sources of bias	5	Methods, Paragraph 3
Study size	10	Explain how the study size was arrived at	4	Methods, Paragraph 2
Quantitativevariables	11	Explain how quantitative variables were handled in the analyses. If applicable, describe which groupings were chosen and why	4–5	Methods, Paragraph 6
Statistical methods	12	(a) Describe all statistical methods, including those used to control for confounding		
		(b) Describe any methods used to examine subgroups and interactions		
		(c) Explain how missing data were addressed		
		(d) Cohort study—If applicable, explain how loss to follow-up was addressed Case–control study—If applicable, explain how matching of cases and controls was addressed Cross-sectional study—If applicable, describe analytical methods taking account of sampling strategy	4–5	Methods, Paragraph 6
		(e) Describe any sensitivity analyses		
Results				
Participants	13 *	(a) Report numbers of individuals at each stage of study—e.g., numbers potentially eligible, examined for eligibility, confirmed eligible, included in the study, completing follow-up, and analysed		
		(b) Give reasons for non-participation at each stage		
		(c) Consider use of a flow diagram		
Descriptive data	14 *	(a) Give characteristics of study participants (e.g., demographic, clinical, social) and information on exposures and potential confounders	5–7	Results, Paragraph 1, Table 1, Figure 1 and Figure 2
		(b) Indicate number of participants with missing data for each variable of interest		
		(c) *Cohort study*—Summarise follow-up time (e.g., average and total amount)		
Outcome data	15 *	*Cohort study*—Report numbers of outcome events or summary measures over time		
		*Case–control study—Report numbers in each exposure category, or summary measures of exposure*		
		*Cross-sectional study—Report numbers of outcome events or summary measures*	*10–13*	*Results, Paragraphs 2–5, Table 2, Table 3, Table 4, Table 5, Table 6, Table 7 and Table 8, Figure 1, Figure 2 and Figure 3*
Main results	16	(*a*) Give unadjusted estimates and, if applicable, confounder-adjusted estimates and their precision (e.g., 95% confidence interval). Make clear which confounders were adjusted for and why they were included		
		(*b*) Report category boundaries when continuous variables were categorized		
		(*c*) If relevant, consider translating estimates of relative risk into absolute risk for a meaningful time period		
Other analyses	17	Report other analyses done—e.g., analyses of subgroups and interactions, and sensitivity analyses		
Discussion				
Key results	18	Summarise key results with reference to study objectives	14	Discussion, Paragraph 1
Limitations	19	Discuss limitations of the study, taking into account sources of potential bias or imprecision. Discuss both direction and magnitude of any potential bias	15	Discussion, Paragraph 9
Interpretation	20	Give a cautious overall interpretation of results considering objectives, limitations, multiplicity of analyses, results from similar studies, and other relevant evidence	16	Discussion, Paragraph 10
Generalisability	21	Discuss the generalisability (external validity) of the study results	16	Discussion, Paragraph 9–10
Other information				
Funding	22	Give the source of funding and the role of the funders for the present study and, if applicable, for the original study on which the present article is based	16	Declaration, Paragraph 5

* Give information separately for cases and controls in case–control studies and, if applicable, for exposed and unexposed groups in cohort and cross-sectional studies. Note: An Explanation and Elaboration article discusses each checklist item and gives methodological background and published examples of transparent reporting. The STROBE checklist is best used in conjunction with this article (freely available on the Web sites of PLoS Medicine at http://www.plosmedicine.org/, Annals of Internal Medicine at http://www.annals.org/, and Epidemiology at http://www.epidem.com/). Information on the STROBE Initiative is available at www.strobe-statement.org.

## Appendix B. Questionnaires and Scales

**Section A: The Perceived Stress Scale (PSS)**

1. Please answer how often you felt or thought a certain way during the last month

The last month, how often have you been upset because of something that happened unexpectedly?

- Never
- Almost never
- Sometimes
- Fairly often
- Very often

In the last month, how often have you felt that you were unable to control the important things in your life?

- Never
- Almost never
- Sometimes
- Fairly often
- Very often

In the last month, how often have you felt nervous and stressed?

- Never
- Almost never
- Sometimes
- Fairly often
- Very often

In the last month, how often have you felt confident about your ability to handle your personal problems?

- Never
- Almost never
- Sometimes
- Fairly often
- Very often

In the last month, how often have you felt that things were going your way?

- Never
- Almost never
- Sometimes
- Fairly often
- Very often

In the last month, how often have you found that you could not cope with all the things you had to do?

- Never
- Almost never
- Sometimes
- Fairly often
- Very often

In the last month, how often have you been able to control irritations in your life?

- Never
- Almost never
- Sometimes
- Fairly often
- Very often

In the last month, how often have you felt that you were on top of things?

- Never
- Almost never
- Sometimes
- Fairly often
- Very often

In the last month, how often have you been angered because of things that happened that were outside of your control?

- Never
- Almost never
- Sometimes
- Fairly often
- Very often

In the last month, how often have you felt difficulties were piling up so high that you could not overcome them?

- Never
- Almost never
- Sometimes
- Fairly often
- Very often

**Section B: Brief patient health questionnaire (Brief PHQ)**

Over the last 2 weeks, how often have you been bothered by any of the following problems

Little interest or pleasure in doing things:

- Not at all
- Several days
- More than half the days
- Nearly every day

Feeling down, depressed, or hopeless:

- Not at all
- Several days
- More than half the days
- Nearly every day

Trouble falling or staying asleep, or sleeping too much:

- Not at all
- Several days
- More than half the days
- Nearly every day

Feeling tired or having little energy:

- Not at all
- Several days
- More than half the days
- Nearly every day

Poor appetite or overeating:

- Not at all
- Several days
- More than half the days
- Nearly every day

Feeling bad about yourself, or that you are a failure, or have let yourself or your family down:

- Not at all
- Several days
- More than half the days
- Nearly every day

Trouble concentrating on things, such as reading the newspaper or watching television:

- Not at all
- Several days
- More than half the days
- Nearly every day

Moving or speaking so slowly that other people could have noticed. Or the opposite—being so fidgety or restless that you have been moving around a lot more than usual:

- Not at all
- Several days
- More than half the days
- Nearly every day

Thoughts that you would be better off dead, or of hurting yourself in some way:

- Not at all
- Several days
- More than half the days
- Nearly every day

In the last 4 weeks, have you had an anxiety attack (suddenly feeling fear or panic)?

- No
- Yes

If yes please answer the following questions

Has this ever happened before?

- Yes
- No

Do some of these attacks come suddenly out of the blue—that is, in situations where you don’t expect to be nervous or uncomfortable?

- Yes
- No

Do these attacks bother you a lot or are you worried about having another attack?

- Yes
- No

During your last bad anxiety attack, did you have symptoms like shortness of breath, sweating, your heart racing or pounding, dizziness or faintness, tingling or numbness, or nausea or upset stomach?

- Yes
- No

If you checked off any problems on this questionnaire so far, how difficult have these problems made it for you to do your work, take care of things at home, or get along with other people?

- Not difficult at all
- Somewhat difficult
- Very difficult
- Extremely difficult

For Women Only Question about menstruation, pregnancy, and childbirth

Which best describes your menstrual periods?

- Periods are unchanged
- No periods because pregnant or recently gave birth
- Periods have become irregular or changed in frequency, duration, or amount
- No periods for at least a year
- Having periods because taking hormone replacement (estrogen) therapy or oral contraceptives

During the week before your period starts, do you have a serious problem with your mood—like depression, anxiety, irritability, anger, or mood swings?

- Yes
- No (or does not apply)

If YES, do these problems go away by the end of your period?

- Yes
- No

Have you given birth within the last 6 months?

- Yes
- No (or does not apply)

Have you had a miscarriage within the last 6 months?

- Yes
- No (or does not apply)

Are you having difficulty getting pregnant?

- Yes
- No (or does not apply)

**Section C: Social Support Questionnaire (Academic Adaptation)**

Please indicate how much you agree with each statement:

- I can rely on my professors when things get difficult during my studies.
- My professors are willing to listen to my academic problems.
- My professors offer help when I am under pressure.
- I feel supported by my professors during challenging times.

Response options for each:

- Not at all
- A little
- Mostly
- Absolutely

**Section D: Group Identification Scale**

Please indicate how much you agree with each of the following statements:

- I identify strongly with other students in my study program.
- I feel a bond with other students in my study program.
- I feel solidarity with other students in my study program.
- I feel a connection to other students in my study program.

Response options for each:

- Strongly disagree
- Disagree
- Somewhat disagree
- Neither agree nor disagree
- Somewhat agree
- Agree
- Strongly agree

**Section E: Subjective Social Status Scale**

Below is a visual ladder representing where people stand in society. At the top of the ladder (100) are the people who are best off-those who have the most money, most education, and the most respected jobs. At the bottom (0) are the people who are worst off-who have the least money, least education, and the least respected jobs or no job.
