# Supplementary material for: Perceived Social Support, Study-Related Stress, and Depressive Symptoms in Saudi Medical Students: A Cross-Sectional Study
Source: Healthcare (Basel). 2026 Jun 23;14(13):1816. doi: 10.3390/healthcare14131816 (PMC13360849; doi:10.3390/healthcare14131816)
Supplement: Supplementary file 1 [file healthcare-14-01816-s001.zip › healthcare-4303434-supplementary.pdf]

Supplementary Material

Supplementary Figure S1. STROBE participant-flow diagram.

Of 1,654 eligible medical students invited through institutional student channels, 511 was the pre-specified target sample size. A total of 367 students provided informed consent and completed all required items of the online questionnaire and were included in the final analysis (22.2% of those invited; 71.8% of the pre-specified target). Because completion of every item was required for submission, there were no partially completed questionnaires among the analysed records; students who opened the link but did not consent or did not complete all items were not captured in the final dataset. The flow is summarised below.

Eligible medical students invited (n = 1,654)

↓

Pre-specified target sample size (n = 511)

↓

Consented and completed all items — included in analysis (n = 367) 22.2% of invited; 71.8% of target

Note. Item completion was mandatory for submission; complete-case analysis was therefore equivalent to the full analysed sample. Self-selection and non-response among the non-participating invitees could not be characterised and are addressed in the Limitations.

Supplementary Table S1. Item-level responses to the Perceived Stress Scale (PSS-10) (n = 367).

PSS-10 item (past month)	Never	Almost never	Sometimes	Fairly often	Very often
Been upset because of something that happened unexpectedly	29 (7.9%)	61 (16.6%)	170 (46.3%)	70 (19.1%)	37 (10.1%)
Felt unable to control the important things in your life	45 (12.3%)	86 (23.4%)	135 (36.8%)	69 (18.8%)	32 (8.7%)
Felt nervous and stressed	22 (6.0%)	55 (15.0%)	132 (36.0%)	89 (24.3%)	69 (18.8%)
Felt confident about handling personal problems (R)	39 (10.6%)	97 (26.4%)	155 (42.2%)	52 (14.2%)	24 (6.5%)
Felt that things were going your way (R)	18 (4.9%)	76 (20.7%)	174 (47.4%)	71 (19.3%)	28 (7.6%)
Found you could not cope with all the things you had to do	33 (9.0%)	85 (23.2%)	153 (41.7%)	65 (17.7%)	31 (8.4%)
Been able to control irritations in your life (R)	32 (8.7%)	91 (24.8%)	153 (41.7%)	64 (17.4%)	27 (7.4%)
Felt that you were on top of things (R)	23 (6.3%)	61 (16.6%)	155 (42.2%)	95 (25.9%)	33 (9.0%)
Been angered by things outside of your control	36 (9.8%)	80 (21.8%)	144 (39.2%)	70 (19.1%)	37 (10.1%)
Felt difficulties were piling up so high you could not overcome them	42 (11.4%)	98 (26.7%)	130 (35.4%)	64 (17.4%)	33 (9.0%)

Note. (R) denotes positively worded items that were reverse-coded before computing the total PSS-10 score (items 4, 5, 7, and 8). Corrected PSS-10: mean 20.19 ± 6.21; Cronbach's α = 0.79.

Supplementary Table S2. Item-level responses to the Patient Health Questionnaire (PHQ-9) (n = 367).

PHQ-9 item (past two weeks)	Not at all	Several days	Nearly half the days	Almost every day
Little interest or pleasure in doing things	77 (21.0%)	167 (45.5%)	91 (24.8%)	32 (8.7%)
Feeling down, depressed, or hopeless	100 (27.2%)	164 (44.7%)	75 (20.4%)	28 (7.6%)

PHQ-9 item (past two weeks)	Not at all	Several days	Nearly half the days	Almost every day
Trouble falling or staying asleep, or sleeping too much	99 (27.0%)	147 (40.1%)	80 (21.8%)	41 (11.2%)
Feeling tired or having little energy	51 (13.9%)	169 (46.0%)	97 (26.4%)	50 (13.6%)
Poor appetite or overeating	124 (33.8%)	132 (36.0%)	62 (16.9%)	49 (13.4%)
Feeling bad about yourself / a failure / let self or family down	134 (36.5%)	118 (32.2%)	65 (17.7%)	50 (13.6%)
Trouble concentrating on things	147 (40.1%)	113 (30.8%)	76 (20.7%)	31 (8.4%)
Moving/speaking slowly, or being fidgety/restless	189 (51.5%)	107 (29.2%)	48 (13.1%)	23 (6.3%)
Item 9: Thoughts of being better off dead, or of hurting yourself	209 (56.9%)	89 (24.3%)	45 (12.3%)	24 (6.5%)

Note. PHQ-9: mean 9.45 ± 5.58; Cronbach's α = 0.84; 177 (48.2%) screened positive for clinically significant depressive symptoms (PHQ-9 ≥ 10). Item 9 is a screening item and not a clinical diagnosis of suicidality; 158 (43.1%) endorsed it at any frequency. Counselling and helpline information was provided to all participants (see Methods).

Supplementary Table S3. Descriptive responses to reproductive-health screening items (unverified; reported for transparency only).

Caution: The childbirth, miscarriage, and difficulty-conceiving items below were administered to all 367 participants using a combined "No (does not apply)" response option. The resulting affirmative proportions are implausibly high for a predominantly single student sample and most likely reflect a skip-logic or item-coding problem that could not be verified from the anonymised dataset. These items were *not* used in any analysis or interpretation and are presented here for transparency only. Only the premenstrual-mood items, reported on the female denominator (n = 189), are referred to in the main text.

Reproductive-health screening item	Response	n (%)
Serious mood problem in the week before period (depression, anxiety, irritability, anger, mood swings)	Yes	140 (38.1%)
	No / does not apply	227 (61.9%)
If yes, do these problems resolve by the end of the period?	Yes	130 (35.4%)
	No	71 (19.3%)
Given birth within the last 6 months †	Yes	50 (13.6%)
	No / does not apply	317 (86.4%)
Miscarriage within the last 6 months †	Yes	41 (11.2%)
	No / does not apply	179 (48.8%)
Difficulty getting pregnant †	Yes	41 (11.2%)
	No / does not apply	174 (47.4%)

Note. Percentages are of the full sample (n = 367). † These items are considered unreliable owing to a probable skip-logic/coding error (the row totals for the affirmative and "no/does not apply" options do not sum to 367, indicating inconsistent routing) and are reported for transparency only; they were excluded from all analyses. Premenstrual-mood findings cited in the main text are computed on female participants (n = 189): 110 (58.2%) reported premenstrual mood disturbances and 97 (51.3% of female participants) reported resolution after menstruation.
